# A Pilot Trial of Pioglitazone HCl and Tretinoin in ALS: Cerebrospinal Fluid Biomarkers to Monitor Drug Efficacy and Predict Rate of Disease Progression

**DOI:** 10.1155/2012/582075

**Published:** 2012-06-28

**Authors:** Todd D. Levine, Robert Bowser, Nicole C. Hank, Stephen Gately, Dietrich Stephan, David S. Saperstein, Kendall Van Keuren-Jensen

**Affiliations:** ^1^Phoenix Neurological Associates, 5090 N 40th Street Suite 250, Phoenix, AZ 85018, USA; ^2^Division of Neurology, Barrow Neurological Institute, 350 W Thomas Road, Phoenix, AZ 85013, USA; ^3^Division of Neurogenomics, Translational Genomics Research Institute (TGen), 445 N 5th Street, Phoenix, AZ 85004, USA; ^4^Silicon Valley Biosystems, 3000 Sand Hill Road, Menlo Park, CA 94025, USA

## Abstract

*Objectives*. To determine if therapy with pioglitazone HCl and tretinoin could slow disease progression in patients with ALS. Levels of tau and pNFH in the cerebrospinal fluid were measured to see if they could serve as prognostic indicators. *Methods*. 27 subjects on stable doses of riluzole were enrolled. Subjects were randomized to receive pioglitazone 30 mg/d and tretinoin 10 mg/BID for six months or two matching placebos. ALSFRS-R scores were followed monthly. At baseline and at the final visit, lumbar punctures (LPs) were performed to measure cerebrospinal fluid (CSF) biomarker levels. *Results*. Subjects treated with tretinoin, pioglitazone, and riluzole had an average rate of decline on the ALSFRS-R scale of −1.02 points per month; subjects treated with placebo and riluzole had a rate of decline of −.86 (*P* = .18). Over six months of therapy, CSF tau levels decreased in subjects randomized to active treatment and increased in subjects on placebo. Further higher levels of pNF-H at baseline correlated with a faster rate of progression. *Conclusion*. ALS patients who were treated with tretinoin and pioglitazone demonstrated no slowing on their disease progression. Interestingly, the rate of disease progression was strongly correlated with levels of pNFH in the CSF at baseline.

## 1. Introduction

Amyotrophic lateral sclerosis (ALS) is a devastating progressive neurodegenerative disease. ALS affects people of all ages and both sexes with a reported annual incidence of 1-2 in 100,000. 90% of the cases of ALS occur sporadically, while 10% of patients with ALS have a family history of the disease. The course of ALS is progressive and the majority of the patients succumb within 2–5 years from disease onset. At present, there is no effective therapy for ALS, nor is there a single known cause. After several decades of trials, only one drug with modest disease modifying efficacy, riluzole, has been developed. Therefore, it is imperative for clinical trials to be designed and analyzed quickly. With the disease being rapidly fatal, eligible patients are difficult to enroll in studies, yet the need for compassionate care is essential.

Recent discoveries have identified several single-nucleotide polymorphisms (SNPs) in the anaplastic lymphoma kinase (ALK) gene which have been associated with sporadic ALS [1, 2]. In addition, reduced levels of ALK mRNA have been reported in ALS patients. In 2003, Dangond et al. used spinal cord tissue and microarray analysis and observed a 9-fold downregulation of ALK mRNA in ALS patients [3]. They further substantiated this finding by verifying the change in ALK expression with real-time quantitative RT-PCR. Midkine (MK), a ligand for ALK, was also found to be decreased in patients with sporadic ALS [4].

The expression of MK is induced by retinoic acid signaling, and in addition to activating ALK, MK has the ability to activate neurite outgrowth and increase angiogenesis. It has also been shown that retinoic acid inducible genes improve survival in the G93A-SOD1 transgenic mouse model of ALS [5]. Further, two of the best-studied and most beneficial gene therapies in animal models of ALS, insulin growth factor (IGF), and vascular endothelial growth factor (VEGF), are induced by retinoic acid.

The importance of retinoic acid responsive genes in modulating spinal motor neuron degeneration has been demonstrated in rodent models [5]. It was demonstrated that astrocytosis, accumulation of neurofilament, motor neuron loss, and a significant loss of motor neuron retinoic acid receptor expression were observed in the lumbar spinal cord of vitamin A deficient animals. Recent findings of the role of retinoid signaling in the pathogenesis of ALS have gained momentum with the development of gene expression analysis in spinal cord from animal models and humans with disease [6]. Retinoid signaling, while crucial in the development and maintenance of the central nervous system (CNS), may also contribute to regenerative mechanisms that occur after CNS injury or disease to mediate plasticity and repair. Additional evidence supporting the role of retinoid signaling comes from proteomic studies of cerebrospinal fluid (CSF). Reduced levels of transthyretin (TTR) and increased posttranslational modifications to TTR have been detected in the CSF of ALS patients [7]. TTR functions to transport and deliver retinol to cells of the CNS. Altered protein levels for multiple members of the retinoid signaling pathway have been detected in spinal cord tissue of ALS patients [4]. Data also suggests that stimulating retinoic acid receptors (RARs) are neuroprotective and therefore pharmacologic agents that target these nuclear receptors may be of value in slowing the progression of ALS [8].

 Retinoic acids have been studied extensively in various models of the injured nervous system. These studies have shown that retinoic acids may have three distinct, important roles in ALS. (1) Retinoids may be neuroprotective and support axonal growth. (2) They may modulate the inflammatory reaction by microglia and macrophages. (3) They may regulate glial cell differentiation [9]. These findings suggest that retinoid signaling might slow the progression of sporadic ALS.

Another potential etiology of ALS, as well as other neurodegenerative diseases, is neuroinflammation. In the last decade, the neuroprotective properties of proliferator-activated receptor gamma (PPAR gamma) agonists have received increasing attention and have been examined in a number of preclinical models of neurodegenerative conditions, including Parkinson's disease, Alzheimer's disease, cerebral ischemia, ALS, and spinal cord injury. These diseases share an excess of neurotoxic, proinflammatory immune responses as compared to anti-inflammatory microglia or T suppressor cell responses [10]. Therefore, therapeutic strategies designed to modulate microglial activation, reinstating the physiological shift toward less neurotoxic phenotypes, may represent a neuroprotective goal. PPAR gamma agonists act as potent anti-inflammatory drugs and have been studied in G93A-SOD1 transgenic mice, a mouse model of ALS. These studies demonstrate that pioglitazone HCl-treated transgenic mice have improved muscle strength and body weight, exhibit a delayed disease onset, and survive significantly longer than nontreated G93A-SOD1 mice. Quantification of motor neurons of the spinal cord at day 90 revealed complete neuroprotection by pioglitazone HCl, whereas non-treated G93A-SOD1 mice had lost 30% of motor neurons. This was paralleled by preservation of the median fiber diameter in the quadriceps muscle, indicating not only morphological, but also functional protection of motor neurons by pioglitazone HCl. Activated microglia were significantly reduced at sites of neurodegeneration in pioglitazone HCl treated G93A-SOD1 mice, as were the protein levels of cyclooxygenase-2 and inducible nitric oxide syntheses [11].

While a PPAR gamma and a retinoic acid agent could be studied individually, a drug cocktail may offer the best chance of attaining a significant reduction in disease progression, utilizing currently available FDA-approved agents. Therefore, a trial of riluzole, in combination with pioglitazone HCl, a PPAR gamma agonist, and tretinoin, a retinoid, was undertaken.

For the past few years, there has been growing evidence that throughout the course of ALS, proteins are released from injured/dying axons and cell bodies that can be detected in the CSF. Developing biomarkers for ALS has emerged as one of the most urgent needs in the search for effective treatments, because these proteins may provide prognostic indicators for rate of disease progression, or they may be used to monitor therapeutic efficacy of drugs that reduce motor neuron injury and/or cell death. Of these proteins, cytoskeletal proteins including phosphorylated neurofilament heavy chain (pNFH) and tau have been shown to be elevated in the CSF of neurodegenerative diseases and proposed as biomarkers for ALS [12–15] and neurofilament aggregates have been observed in spinal cord motor neurons of ALS patients [16]. Levels of pNFH were shown to be significantly increased in the CSF of ALS patients when compared to disease mimics [17, 18]. Therefore, we also measured the levels of these candidate biomarkers before treatment and after six months of drug therapy.

## 2. Methods and Materials

### 2.1. Subjects

Twenty-eight subjects, who met the El Escorial criteria for probable or definite ALS, between the ages of 18 and 85 and were on a stable dose of Rilutek for at least 30 days prior to the start of the study, were screened. Women of childbearing potential were using an effective method of birth control and had a negative pregnancy test prior to randomization. Subjects who had a history of liver disease, severe renal failure, diabetes, coronary heart disease, clinically significant EKG abnormality at screening or intolerance to Riluzole, or any other comorbid condition which would make completion of the trial unlikely or a FVC of less than 70% were excluded. All procedures were approved by the Western Institutional Review Board (WIRB) and were conducted with the understanding and consent of all subjects. Informed consents were obtained from all individuals in accordance with institutional review board requirements prior to the start of any study-related procedures at screening.

### 2.2. Study Procedures

Prior to enrollment, potential subjects were evaluated, screened, and consented. A medical history was completed and a physical and neurological exam was performed. An EKG was performed to rule out significant cardiac abnormalities and a FVC test was completed for eligibility. Subjects who met all inclusion criteria and met no exclusionary criteria underwent a lumbar puncture and 6 cc of CSF were collected for analyzing specific proteins such as tau and pNF-H at screening. Subjects were also required to fast prior to screening and prior to every subsequent visit. This ensured accurate results for glucose and lipid testing. Amylase, lipase, and a comprehensive metabolic panel were also monitored monthly. Eligible subjects came back for randomization 2 months after screening. This two-month run-in phase was designed to see if we could determine an individual patient's baseline rate of progression. Subjects randomized to drug received 30 mg/day of pioglitazone HCl and 10 mg BID of Tretinoin. Subjects randomized to placebo received matching placebos for pioglitazone HCl and tretinoin. Drug and matching placebo were manufactured by The Apothecary Shop in Scottsdale, Arizona. An unblinded member of PNA's study team randomized subjects based on screening number and dispensed the appropriate medication to subjects. The PI, study coordinator and the patient were blinded during treatment. Subjects who experienced intolerable side effects abated the tretinoin but continued taking pioglitazone HCl (*N* = 5). 2 patients on placebo reported side effects and had the placebo matching the tretinoin stopped. Subjects were seen 8 weeks after screening for randomization then again at months 1, 2, 3, 4, and 6. The primary outcome measurement, ALSFRS revised (ALSFRS-R), was administered and assessed by the same rater at each visit to insure reproducibility and reduce variability. The last recorded ALSFRS-R score was subtracted from their baseline ALSFRS-R score and was divided by the number of months that the subjects were on treatment to determine a monthly change in ALSFRS-R score. Secondary outcome measures were evaluated at baseline then again at the final visit (after 6 months of treatment), which included lumbar punctures for assessment of protein biomarkers of axonal injury.

### 2.3. CSF Collection and Analysis

Lumbar punctures were performed at baseline and the final visit. Samples were collected at a similar time of the day for all subjects. 6 ccs of cerebrospinal fluid (CSF) were collected into a low-bind tube and frozen in a −80°C freezer for storage. All samples were coded to maintain patient confidentiality. Total protein concentrations were determined using the BCA Protein Assay Kit (Thermo Scientific, Waltham, MA). Levels of candidate biomarkers were determined using commercial ELISA kits to total tau (Invitrogen, Carlsbad, CA) and phosphorylated neurofilament heavy chain (pNF-H) (BioVendor Research and Diagnostic Products, Candler, NC) following manufacturer instructions. All samples were analyzed in triplicate within each experiment, and all experiments were performed at least twice on separate days.

Patient disease progression was monitored by decline in the revised ALS functional rating scale (ALSFRS-R) and correlated to CSF protein levels using the Pearson correlation test. These correlations were stratified into placebo, tretinoin plus pioglitazone HCl, and pioglitazone HCl-alone treatment groups. For group comparisons, nonparametric Mann-Whitney *t*-test was used to determine statistical significance, followed by paired *t*-test for pairwise comparisons within individual patients between baseline and final values. For all data analysis, we set a significance level of *P* < 0.05. All statistical analysis was performed using GraphPad Prism 5.0 software (GraphPad Software Inc. La Jolla, CA).

## 3. Results

### 3.1. Study Population

Twenty-eight subjects were screened, but only 27 enrolled in this double blinded, placebo controlled study (12 females and 15 males). Subjects were randomized in a 2 : 1 fashion to tretinoin and pioglitazone HCL or placebo. Subjects who were randomized to the drug arm received tretinoin at 10 mg BID and pioglitazone HCL at 30 mg QD. Subjects who were randomized to the placebo arm received matching placebos for both medications. Baseline demographics and functional indices are as noted: average FVC was 82%, average time from symptom onset was 5 months, the average baseline ALSFRS-R score was 37, and the mean age was 58. There was no significant difference between the placebo and the active arm in any of these demographic features. The average time from symptom to onset to study entry was 18 months for placebo and 24 months for the active arm.

### 3.2. Effect of Therapy on Disease Progression

There were 22 subjects who completed more than three months of therapy and these were all included in an intent-to-treat analysis. Of these 22 subjects, 16 were randomized to drug and 6 to placebo. There were five subjects in the active arm who experienced intolerable side effects (1 extreme dry skin, 3 felt they were progressing faster, and 1 had increased fatigue). All five subjects stopped taking the pill corresponding to the tretinoin and two of the five subjects' symptoms abated shortly after discontinuation. 3 patients felt they were progressing more rapidly on therapy and this continued even after stopping all study medication. These patients had a baseline rate of progression in the run-in phase of 1.83, and once starting therapy, their monthly change in ALSFRS-R was 1.75 (*P* = 0.71). The 16 subjects who were on active drug and completed at least 3 months of therapy lost on average −1.02 compared to their baseline rate of progression of −1.13 (*P* = 0.13). The 6 subjects on placebo averaged a loss of −0.86 compared to their baseline rate of progression of 1.11 (*P* = 0.38).

### 3.3. CSF Biomarkers

**Figure 1:**
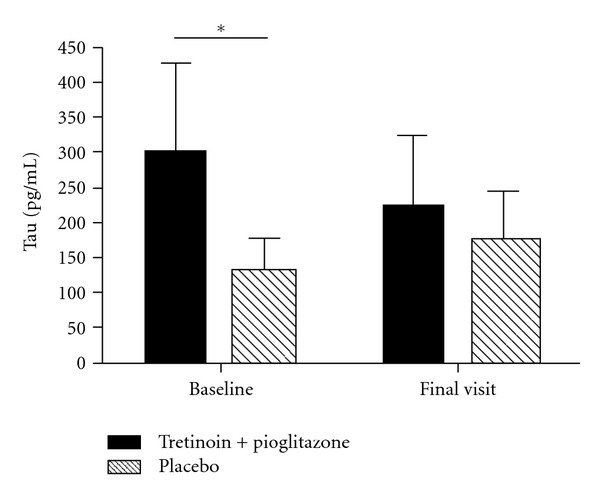
Levels of tau in CSF of patients in active arm versus placebo arm at baseline and after six months of therapy. (*Significant difference in levels of tau at baseline between the two groups, *P* = 0.02 by Mann-Whitney *t*-test). Error bars denote standard deviation.

The baseline levels of tau in the CSF differed significantly between the two groups. The average tau level for the active arm was 300.7 pg/mL (*n* = 10) as compared to 126.5 pg/mL for the placebo arm (*n* = 4) (Figure 1). After six months of therapy the active arm (*n* = 10) demonstrated a 26% decline in the tau values to 223.9 pg/mL (*P* = 0.22), whereas the placebo arm (*n* = 4) demonstrated a 29% increase in tau values (*P* = 0.48). We did not detect a significant change within individuals over time using a paired *t*-test (data not shown). There was also no relationship between the levels of tau and patient rate of progression.

**Figure 2:**
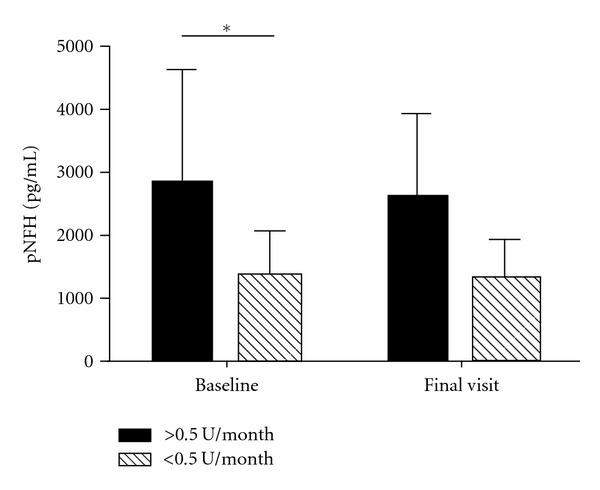
Average pNFH levels in patients who lost <0.5 ALSFRS-R points per month compared to patients who lost >0.5 ALFRS-R points per month. (*Significant difference between the slow and fast progressors, *P* = 0.03 by Mann-Whitney *t*-test). Error bars denote standard deviation.

The CSF levels of pNFH did not differ between the active and placebo groups and there was no significant change during the course of therapy for individual patients (data not shown). However there was a strong statistical relationship between the baseline value of pNFH and the rate of disease progression (Figure 2). Patients who progressed faster than 0.5 points per months on the ALSFRS-R had an average level of 2860 pg/mL compared to patients who progressed less than 0.5 points per month who averaged 1331 pg/mL (*P* = 0.03).

### 3.4. Survival

There were 18 subjects who completed the entire 6 months of therapy. One patient dropped out prior to being randomized, and five subjects withdrew after at least two months of therapy. Of the five who withdrew, 3 were on treatment and 2 were on placebo. During the course of the study, 4 subjects died (2 on active drug and 2 on placebo). Three of the four subjects died of respiratory complications and one committed suicide (they had no prior history of depression or suicidal ideation). The average baseline ALSFRS-R score for the subjects who died was 33, which was lower than the average baseline value (38) for those that completed the study (*P* = 0.222).

### 3.5. Compliance and Safety

Compliance was measured through drug accountability. All subjects were required to return unused medication at each visit. Drug was accounted for by the amount of drug returned subtracted from drug dispensed based on how much drug should have been taken. Tretinoin administered at 10 mg bid was tolerated by 69% and pioglitazone HCl was tolerated by 88% of the subjects randomized to active treatment. There was a 19% dropout rate due to adverse events or intolerability. A DSMB board met shortly after the first four patients were randomized, then every six months thereafter to assure safety, tolerability, and study compliance. Other than one suicide, no other serious events were recorded. At the interim analysis, the majority of subjects tolerated the medication well. Shortly after the last patient completed 1 month on study drug, the FDA reported that the use of pioglitazone HCl increased the rate of bladder cancer. Subjects who were actively taking pioglitazone HCl were contacted and were given the option to withdraw or discontinue use. Everyone continued on treatment, but we felt, in the best interest of others, to stop enrollment at 28 instead of the planned 30.

## 4. Discussion

This initial phase II trial of tretinoin and pioglitazone HCl used in combination with riluzole showed no significant effect on the rate of disease progression. One of the weaknesses of this study was the small sample size. However, the trial was designed to see if there was a significant and dramatic effect on disease progression. This type of a dramatic treatment effect is the primary goal in ALS trials, as another agent with minimal efficacy is not desirable. If some treatment effect had been found, then this trial would have formed the basis for a larger phase II/III trial. Unfortunately, the results demonstrated lack of efficacy with tretinoin and pioglitazone HCl. Another weakness is the use of two experimental drugs and the use of riluzole in all subjects. These were compromises that were intentionally made to address limitations such as the cost of conducting a larger trial, the difficulty of recruiting subjects, and the difficulty of asking patients with ALS to forgo the only available therapy.

Among the 16 subjects randomized to active treatment who completed more than 3 months of therapy, the average decline in ALSFRS-R was −1.02 points per month. This was higher than the rate of decline in subjects randomized to placebo (−0.86); however, this was not significant (*P* = 0.63). There was also no significant difference between the rate of progression observed during the two-month run-in phase and the rates of progression seen in the placebo and active arms during therapy.

The results from our CSF biomarker studies suggest a correlation between the baseline level of pNFH and disease progression. Subjects who progressed at rates faster than −0.5 points per month on the ALSFRS-R scale had levels of pNF-H that averaged 2860 pg/mL, while subjects who progressed at less than −0.5 points per month averaged 1331 pg/mL (*P* = 0.03) (Figure 2). Our results suggest that elevated levels of pNFH in the CSF may serve as a candidate biomarker for injury or death of motor neurons in patients with ALS. Thus higher CSF levels of pNFH may indicate patients with faster disease progression, a finding that mirrors results of a prior clinical study with memantine [19].

Subjects randomized to combination therapy had a nonstatistically significant decrease in tau CSF levels during the course of treatment (an average decrease of 76.8 pg/mL) and 26% decrease (*P* = 0.167) compared to subjects on placebo whose levels increased (average increase of 44.91 pg/mL and 29% increase (*P* = 0.412) (Figure 1). These results may be skewed as there was a significant difference in baseline levels of tau between the active and placebo groups, such that the active arm had 2.4 times the amount of tau at baseline. Patients were not stratified based on these levels, thus this difference was purely coincidental. Therefore, the decline in the active arm and the increase in placebo arm may simply be a regression towards the mean. Further, levels of tau did not correlate to the rate of clinical progression as measured by the ALSFRS-R (data not shown).

## 5. Conclusion

In summary, our randomized, placebo-controlled trial combining riluzole with tretinoin and pioglitazone HCl failed to alter clinical disease progression over a 6-month time course. CSF-based biomarkers also failed to exhibit significant changes due to drug treatment, though initial levels of pNFH correlated to rate of disease progression. However, it remains possible that other drug treatments that target these same biochemical pathways may be beneficial for ALS.

## References

[1] Chiò A, Schymick JC, Restagno G, Scholz SW, Lombardo F (2009). A two-stage genome-wide association study of sporadic amyotrophic lateral sclerosis. *Human Molecular Genetics*.

[2] Dunckley T, Huentelman MJ, Craig DW (2007). Whole-genome analysis of sporadic amyotrophic lateral sclerosis. *The New England Journal of Medicine*.

[3] Dangond F, Hwang D, Camelo S (2004). Molecular signature of late-stage human ALS revealed by expression profiling of postmortem spinal cord gray matter. *Physiological Genomics*.

[4] Jiang YM, Yamamoto M, Kobayashi Y (2005). Gene expression profile of spinal motor neurons in sporadic amyotrophic lateral sclerosis. *Annals of Neurology*.

[5] Corcoran J, So PL, Maden M (2002). Absence of retinoids can induce motoneuron disease in the adult rat and a retinoid defect is present in motoneuron disease patients. *Journal of Cell Science*.

[6] Malaspina A, Kaushik N, De Belleroche J (2000). A 14-3-3 mRNA is up-regulated in amyotrophic lateral sclerosis spinal cord. *Journal of Neurochemistry*.

[7] Ryberg H, An J, Darko S (2010). Discovery and verification of amyotrophic lateral sclerosis biomarkers by proteomics. *Muscle and Nerve*.

[8] Kolarcik, Christi L (2010). *Beyond Biomarker Discovery: Retinoid Signaling in Motor Neurons and Amyotrophic Lateral Sclerosis*.

[9] Mey J (2006). New therapeutic target for CNS injury? The role of retinoic acid signaling after nerve lesions. *Journal of Neurobiology*.

[10] Schintu N, Frau L, Ibba M (2009). PPAR-gamma-mediated neuroprotection in a chronic mouse model of Parkinson’s disease. *European Journal of Neuroscience*.

[11] Schütz B, Reimann J, Dumitrescu-Ozimek L (2005). The oral antidiabetic pioglitazone protects from neurodegeneration and amyotrophic lateral sclerosis-like symptoms in superoxide dismutase-G93A transgenic mice. *Journal of Neuroscience*.

[12] Brettschneider J, Petzold A, Süßmuth SD, Ludolph AC, Tumani H (2006). Axonal damage markers in cerebrospinal fluid are increased in ALS. *Neurology*.

[13] Mares J, Kanovsky P, Herzig R (2009). The assessment of beta amyloid, tau protein and cystatin C in the cerebrospinal fluid: Laboratory markers of neurodegenerative diseases. *Neurological Sciences*.

[14] Süssmuth SD, Tumani H, Ecker D, Ludolph AC (2003). Amyotrophic lateral sclerosis: disease stage related changes of tau protein and S100 beta in cerebrospinal fluid and creatine kinase in serum. *Neuroscience Letters*.

[15] Wild EJ, Petzold A, Keir G, Tabrizi SJ (2007). Plasma neurofilament heavy chain levels in Huntington’s disease. *Neuroscience Letters*.

[16] Wong BS, Pan T, Liu T, Li R, Gambetti P, Sy MS (2000). Differential contribution of superoxide dismutase activity by prion protein in vivo. *Biochemical and Biophysical Research Communications*.

[17] Ganesalingam J, An J, Shaw CE, Shaw G, Lacomis D, Bowser R (2011). Combination of neurofilament heavy chain and complement C3 as CSF biomarkers for ALS. *Journal of Neurochemistry*.

[18] Reijn TS, Abdo WF, Schelhaas HJ, Verbeek MM (2009). CSF neurofilament protein analysis in the differential diagnosis of ALS. *Journal of Neurology*.

[19] Levine TD, Bowser R, Hank N, Saperstein D (2010). A pilot trial of memantine and riluzole in ALS: correlation to CSF biomarkers. *Amyotrophic Lateral Sclerosis*.

